# Advances in post-translational and epigenetic modification of hyperuricemia-to-gout progression: mechanisms and therapeutic targets

**DOI:** 10.3389/fcell.2026.1749194

**Published:** 2026-05-29

**Authors:** Wei Fu, Xiangman Xu, Xinze Li, Qian Guo, Peng Liu, Lulu Chen, Wenfei Cheng, Zikuan Zhang, Zilong Chen, Chuanxin Liu

**Affiliations:** 1 Henan Key Laboratory of Rare Diseases, Endocrinology and Metabolism Center, The First Affiliated Hospital, and College of Clinical Medicine of Henan University of Science and Technology, Luoyang, China; 2 Department of Rhinology, The First Affiliated Hospital of Zhengzhou University, Zhengzhou, China; 3 Department of Cardiology, The First Affiliated Hospital of Henan University of Science and Technology, Luoyang, China

**Keywords:** epigenetic modifications, gout, hyperuricemia, post-translational modifications, therapeutic targets

## Abstract

Hyperuricemia (HUA) and gout are endocrine disorders resulting from abnormal uric acid (UA) metabolism, with gout typically developing secondary to HUA and being associated with an exacerbated inflammatory response. Epigenetic modifications and post-translational modifications (PTMs) may contribute to the progression from HUA to gout by modulating the function of UA transporters such as ABCG2 and GLUT9 and involving the NLRP3/IL-β inflammatory axis. However, the specific mechanisms underlying these processes remain incompletely understood. Therefore, this review systematically examines recent research on epigenetic modifications, such as methylation, lactylation, and crotonylation, as well as PTMs including succinylation, phosphorylation, glycosylation, ubiquitination, and acetylation in this process, with the aim of identifying potential therapeutic targets for these two diseases.

## 1 Introduction

HUA is a disorder of purine metabolism caused by excessive production and/or inadequate excretion of UA, characterized by elevated serum uric acid (SUA) levels (Nakayama et al., 2017; Johnson et al., 2003; Li et al., 2020). Gout, an inflammatory arthritis, often follows HUA and is caused by the deposition of monosodium urate (MSU) in and/or around the joints. Its diagnostic criteria are microscopic confirmation of gouty material or synovial fluid (Bardin and Richette, 2014; Pascual and Jovaní, 2005). Both can lead to conditions such as hypertension (Lee et al., 2015; Grayson et al., 2011), Parkinson’s disease (Pakpoor et al., 2015; Seifar et al., 2022), stroke (Kim et al., 2009; Latourte et al., 2021) and diabetes (Rodríguez et al., 2010; Kodama et al., 2009). Epidemiological studies indicate that HUA and gout are more prevalent in developed countries, affecting men more than women. However, their incidence is rising steadily in developing countries, where they have become a major health burden (Im et al., 2025; Ahn, 2023; Asghari et al., 2024).

PTMs are covalent modifications of proteins, which refers to the addition of various functional groups to amino acid residues through the action of enzymes such as transferases, kinases, and hydrolases (Ramazi and Zahiri, 2021). Approximately 500 types of PTMs have been identified, with common types including phosphorylation, glycosylation, acetylation, ubiquitination and lactylation (Deribe et al., 2010; He et al., 2019). Under physiological conditions, PTMs modulate protein conformation and function, and participate in signal transduction and play a role in metabolic processes to maintain cellular homeostasis (He et al., 2019; Mann and Jensen, 2003). Epigenetic modifications refer to the dynamic and reversible regulation of gene expression through mechanisms such as DNA modifications, non-coding RNAs regulation, chromatin structure modulation, and histone modifications, without altering the DNA sequence. Notably, DNA and RNA methylation, as well as histone modifications, are extensively studied. These modifications regulate physiological processes such as gene expression and the cell cycle by influencing key processes including transcriptional activity, chromatin structure and DNA replication (Du et al., 2026; Tin et al., 2021).

Previous studies have demonstrated that PTMs and epigenetic modifications play significant roles in the pathogenesis of HUA and gout; however, the underlying molecular mechanisms remain unclear. This paper reviews the relevant research progress and summarizes the mechanisms of these factors in the aforementioned diseases, aiming to provide new insights for potential intervention targets.

## 2 HUA and gout

UA is mainly catalyzed in the liver by xanthine oxidase (XO) or xanthine dehydrogenase (XDH) from purines (Hyndman et al., 2016). Under physiological conditions, UA, as a weak organic acid, circulates in the body primarily as the ionized form of urate (particularly MSU) (Maiuolo et al., 2016). While some mammals possess uricase that converts urate into allantoin, this enzyme was lost during human evolution (Johnson et al., 2013). Therefore, once urate is produced in the human body, it must be excreted by the kidneys (about 70%) or intestines, and other ways (about 30%) (Ohno, 2011).

### 2.1 Pathogenesis of HUA

HUA is clinically defined as a UA concentration greater than 420 μmol/L (7 mg/dL) in men and 350 μmol/L (6 mg/dL) in women (Nakayama et al., 2017; Johnson et al., 2003; Li et al., 2020). Its pathogenesis centers on metabolic disorders, primarily characterized by an imbalance caused by excessive UA synthesis and/or insufficient excretion, with reduced excretion being the dominant factor (Wang et al., 2022). Various UA transporters in the intestines and kidneys, including glucose transporter 9 (GLUT9), organic anion transporter 4 (OAT4), and UA transporter 1 (URAT1), enhance UA reabsorption, while the ATP-binding cassette transporter G2 subfamily (ABCG2), sodium-dependent phosphate transporter 1 (NPT1), and organic anion transporters 1 and 3 (OAT1 and OAT3), facilitate UA excretion (DeBosch et al., 2014; Ichida et al., 2012). Abnormalities in the function or structure of these transporters can disrupt UA metabolism. Moreover, normal gut microbiota such as *Lactobacillus* and *Pseudomonas* can synthesize uricase, allantoinase, and other enzymes, which convert UA into urea (El Husseini et al., 2014). However, when the immune system is altered, an increase in inflammatory cytokines can disrupt the gut microbiota balance, leading to abnormal UA excretion (Luissint et al., 2016). Hypoxanthine-guanine phosphoribosyltransferase (HGPRT) recycles excess purines in the body; decreased levels or activity can lead to purine accumulation and increased UA production (López-Cruz et al., 2016). Studies show that high-purine foods such as seafood, dried beans, and beer can increase UA production and HUA risk, while vitamin B12, B6, and folate are linked to a reduced risk (Villegas et al., 2012; Zhang and Qiu, 2018).

### 2.2 Pathogenesis of gout

Gout is characterized by joint warmth, redness, swelling, and severe pain (Neogi et al., 2015). It primarily arises from the innate immune response to deposited MSU crystals. This process is closely associated with the activation of the NLRP3 inflammasome, comprising NLRP3, caspase-1, and the adaptor protein ASC (Martinon et al., 2006). When the SUA levels reach approximately 6.8 mg/dL, MSU crystals are prone to form (Keenan, 2020). Upon phagocytosis by macrophages, MSU crystals activate caspase-1 via the NLRP3 inflammasome, thereby promoting the cleavage of pro-IL-1β into its mature form, IL-1β (Wu et al., 2019; Cobo et al., 2025). Moreover, MSU crystals enhance glucose uptake through GLUT1, thereby stimulating glycolysis and the accumulation of metabolic intermediates, which further amplifies NLRP3 inflammasome activation and IL-1β production (Renaudin et al., 2020). In addition, MSU crystals upregulate JUN expression in macrophages, thereby promoting the transcription of metabolic and inflammatory genes via the JNK-AP-1 signaling pathway, ultimately leading to immunometabolic dysregulation (Cobo et al., 2025; Cobo et al., 2022). As a central mediator of gout, IL-1β binds to its receptors on target cells, triggering downstream signaling cascades and activating pro-inflammatory transcription factors. This leads to the upregulation of cytokines such as tumor necrosis factor (TNF-α), IL-1β and IL-6, as well as chemokines including IL-8, thereby promoting the recruitment and infiltration of neutrophils and other immune cells to sites of MSU crystal deposition and ultimately triggering gouty inflammation (Dalbeth et al., 2021). Previous studies have linked dysregulated fatty acid metabolism, excessive alcohol intake, and high-purine diets to an increased risk of gout (Joosten et al., 2010; Choi et al., 2024a; Choi et al., 2024b).

### 2.3 HUA and gout

**FIGURE 1:**
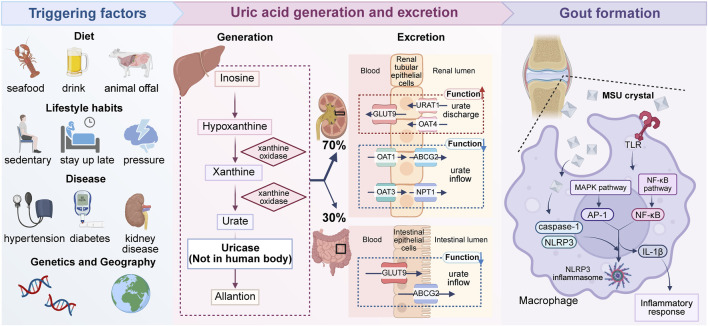
Pathogenic mechanisms from HUA to gout.

HUA is the pathological basis of gout. When the concentration of UA in the body exceeds its solubility threshold, crystals may form and deposit in the joints. Upon phagocytosis by macrophages, MSU crystals activate the inflammasome, thereby triggering an inflammatory response and causing a gout attack (Keyßer, 2020). However, not all patients with HUA develop gout; studies show that only about 36% of HUA patients eventually experience a gout attack (Zhang, 2021). Therefore, HUA is considered a “necessary but insufficient” factor in gout, and this relationship represents a continuous pathological process progressing from metabolic dysregulation to systemic inflammation (Helget and Mikuls, 2022). In addition, as a prerequisite for gout onset, the formation of MSU crystals is influenced not only by HUA but also by low temperature and acidic environments, which can promote crystal formation and precipitation, thereby triggering gout (Wilcox and Khalaf, 1975; Loeb, 1972). The pathogenesis of HUA and gout and their interrelationship are illustrated in Figure 1.

## 3 PTMs and epigenetic modifications

### 3.1 Epigenetic modifications: methylation

Methylation refers to the addition of active methyl groups to DNA, RNA or proteins by methyltransferases, often using S-Adenosylmethionine (SAM) as the methyl donor. DNA methylation often occurs at N6 of adenine, N7 of guanine, and cytosine-phosphoguanine (CpG) dinucleotides, while m6A and m5C are the most representative forms of RNA methylation (Dai et al., 2021; Deaton and Bird, 2011; Chen C.-C. et al., 2013). Methylation is a dynamic enzymatic process, including writing, erasing, and reading catalyzed by methyltransferases, demethylases, and methylation reader proteins respectively (Moore et al., 2013). Methylation of different substrates is mediated by specific methyltransferases. DNA methylation is primarily catalyzed by the Dnmt family (Dnmt1, Dnmt3a, Dnmt3b, Dnmt3L), with Dnmt1 acting as a maintenance methyltransferase that ensures the transmission of methylation patterns during DNA replication (Moore et al., 2013; Pradhan et al., 1999), Dnmt3a and Dnmt3b are structurally and functionally similar and are considered *de novo* methyltransferases and are responsible for catalyzing the methylation of unmethylated DNA to establish new methylation patterns (Moore et al., 2013; Okano et al., 1999). Dnmt3L, a regulatory protein with no catalytic activity, can activate Dnmt3a and Dnmt3b (Moore et al., 2013). The methyltransferases required for RNA methylation are site-specific. For example, m6A methylation is catalyzed by methyltransferase-like 3/14 (METTL3/14), while m5C methylation is catalyzed by NOP2/Sun RNA methyltransferase 2 (NSUN2) (Li Y. et al., 2024).

### 3.2 Epigenetic modifications: histone lactylation (Kla)

Kla refers to a dynamic, reversible modification in which a lactyl group is added to lysine residues on histones under the catalysis of histone acyltransferases (Zhang D. et al., 2019). This modification primarily occurs on histones H3 and H4 (H3, H4), including H3K18la, H3K27la, H3K14la, H3K9la, H3K56la, and H4K12la (Wang et al., 2024). Lactyl groups can also be removed by histone deacetylases (Shang et al., 2022). The accumulation of lactate is a key prerequisite for this process. Kla levels are positively correlated with intracellular lactate concentrations (Li et al., 2022). However, lactate is not the direct donor; rather, it must be converted to lactyl-CoA by the action of a lactyl-CoA-generating enzyme (Zhang X. et al., 2019). Notably, there may be metabolic competition between acetyl-CoA and lactyl-CoA, leading to complex crosstalk between Kla and histone acetylation (Zhang D. et al., 2019). Kla participates in various physiological processes, including cell differentiation, metabolic reprogramming, and inflammatory and immune responses, by influencing the expression of target genes, regulating transcription, and modulating chromatin structure, and also serves as a key link between lactate metabolism and epigenetic modification (Peng and Du, 2025).

### 3.3 Epigenetic modifications: histone crotonylation (Kcr)

Kcr typically refers to the transfer of a crotonyl group to lysine residues on histones, catalyzed by histone acetyltransferases (HATs) (Sabari et al., 2015). This process can occur on H1, H2, H3, and H4, and its levels are regulated by crotonyl-CoA (Wei et al., 2017). The HATs involved in this process primarily include the MYST family, p300/CBP, and the GNAT family, among which p300/CBP exhibits relatively broad acyltransferase activity (Xie et al., 2024). Histone decrotonylases are primarily classified into the NAD^+^-dependent sirtuin family (SIRT1–7) and the HDAC family, with SIRT1–7 and HDAC1–3, 8 being the major histone decrotonylases (Wei et al., 2017). As an evolutionarily conserved epigenetic modification, Kcr is widely found in various eukaryotes and is involved in biological processes such as transcriptional regulation, DNA damage and repair, telomere homeostasis, and the renewal of embryonic stem cells (Xie et al., 2024; Tan et al., 2011).

### 3.4 PTMs: phosphorylation

Protein phosphorylation refers to the transfer of γ-phosphate groups from ATP to substrate proteins through protein kinases, which occurs most frequently at serine (approximately 84%), followed by threonine (approximately 15%) and tyrosine (<1%) (Song et al., 2022; Humphrey et al., 2015). Under physiological conditions, the phosphate group carries two negative charges and can form salt bridges and hydrogen bond networks with amino acids such as arginine and lysine, or interact with the C-terminal nitrogen of the α-helix backbone (Nishi et al., 2014). Protein phosphorylation is a dynamic and reversible process that is finely regulated by the coordinated action of protein kinases and phosphatases (Humphrey et al., 2015). Based on its mode of action, protein phosphorylation can be classified into two modes: molecular switches and molecular potentiometers. The former achieves “on/off” regulation through a single site, while the latter relies on the cumulative effects of multiple sites to achieve gradual regulation (Landry et al., 2014). As one of the earliest identified and most extensively studied PTMs, protein phosphorylation is widespread in cells, occurring in approximately 30% of proteins. By regulating protein structure and function, enzymatic activity, and subcellular localization, as well as through functional interactions with other PTMs, it influences cellular energy metabolism and signal transduction, thereby regulating virtually all cellular physiological processes (Fischer and Krebs, 1955; Cohen, 2002; Cohen, 2000).

### 3.5 PTMs: glycosylation

Protein glycosylation refers to the covalent attachment of monosaccharides or glycans to specific residues of proteins, either via lipid carriers or directly, with N-glycosylation and O-glycosylation being the most common, with less common forms including C-glycosylation, S-glycosylation and P-glycosylation. N-glycosylation often occurs at asparagine residues while O-glycosylation occurs at hydroxyl-containing residues such as serine, threonine and tyrosine (Yang et al., 2024). In eukaryotic cells, glycosylation predominantly occurs on secreted and cell surface proteins, proceeding along the protein synthesis and secretory pathway. This process primarily occurs in the endoplasmic reticulum and Golgi apparatus, and the type of glycosylation is determined by specific initiating enzymes and recognition sequences (Tseng et al., 2020). At the single-protein level, glycosylation is involved in the correct folding of protein conformation, which can affect the activity, stability and solubility of proteins by altering their structure. At the cellular level, proper protein glycosylation contributes to precise protein-protein interactions and facilitates the efficient transmission of signals between cells (Yang et al., 2024).

### 3.6 PTMs: ubiquitination

Protein ubiquitination refers to the process of covalent attachment of ubiquitin to substrate proteins through an enzymatic cascade reaction catalyzed by E1 activating enzymes, E2 conjugating enzymes and E3 ligases. Ubiquitin is an evolutionarily conserved protein of 76 amino acids that differs by only three amino acids between plants, mammals and yeast and is ubiquitously expressed in eukaryotic cells. During ubiquitination, the E1 enzyme activates the glycine at the C-terminus of ubiquitin and forms a thioester bond with a cysteine residue on the E1 enzyme. The ubiquitin is then transferred to the E2 enzyme, and finally, under the mediation of the E3 enzyme, the C-terminus of ubiquitin is linked to the ε-amino group of a lysine residue on the substrate via an isopeptide bond, thereby completing the modification. In some cases, ubiquitin can be transferred directly to the substrate by an E2 enzyme, or it can be attached to the substrate directly by an E3 enzyme, bypassing the E2 enzyme (Hershko and Ciechanover, 1998). Notably, in addition to its conjugation function, E2 enzymes are also involved in various biological processes, such as regulating the cell cycle in fruit flies and mediating DNA repair in yeast (Cenci et al., 1997; Hochstrasser, 1996). It has been shown that ubiquitination regulates several key cellular processes, including the cell cycle, apoptosis, endocytosis and downregulation of receptors, DNA damage repair, proteolysis, intracellular transport and autophagy (Shaid et al., 2013; Popovic et al., 2014).

### 3.7 PTMs: acetylation

Acetylation refers to the transfer of an acetyl group from acetyl-CoA to specific amino acid residues of proteins catalyzed by acetyltransferases, with N-ter acetylation and lysine acetylation (K-acetylation) being the more predominant forms (Polevoda and Sherman, 2002). N-ter acetylation irreversibly occurs at the N-terminal amino acids of proteins, mostly methionine, glycine, threonine, serine and alanine residues, and is prevalent in eukaryotes, occurring co-translationally in ∼85% of eukaryotic proteins, but is less common in prokaryotes (Polevoda and Sherman, 2002; Driessen et al., 1985). K-acetylation is dynamically regulated by lysine acetyltransferases and deacetylases and occurs reversibly in highly structured regions of proteins, such as α-helices and β-sheets. It is widely found in histones, cytoskeletal proteins, and transcription factors (Polevoda and Sherman, 2002; Choudhary et al., 2009). Acetylation participates in several biological processes such as regulation of protein charge, DNA-protein structural stability, transcriptional regulation, tumorigenesis, autoimmune diseases, diabetes and other pathological processes (Drazic et al., 2016).

### 3.8 PTMs: succinylation

Succinylation refers to the addition of a succinyl group to amino acid residues of proteins through enzymatic or non-enzymatic processes; it primarily occurs at lysine residues (Stastna and Van Eyk, 2015). This modification exhibits evolutionary conservation and dynamic reversibility; non-enzymatic succinylation occurs primarily in mitochondria, with substrates derived directly from succinyl-CoA. Enzymatic succinylation is primarily catalyzed by lysine acetyltransferase (KAT2A) and carnitine palmitoyltransferase 1A (CPT1A), with KAT2A primarily mediating H3 succinylation, while the target proteins of CPT1A are predominantly located in the cytoplasm (Wang et al., 2017; Kurmi et al., 2018). Desuccinylation is primarily mediated by SIRT5, which uses nicotinamide adenine dinucleotide (NAD) as a coenzyme to catalyze the removal of the succinyl group (Du et al., 2011). In addition, SIRT7 mediates the desuccinylation of histones (Zhao G. et al., 2022). At physiological pH, lysine succinylation causes the charge of the lysine residue to change from +1 to −1 and significantly affects the protein’s conformation and function due to the introduction of a bulky group (Meng et al., 2019; Weinert et al., 2013). Succinylation plays a crucial role in maintaining normal cellular physiological functions by regulating cellular metabolism, the DNA damage response, and protein structural stability (Sreedhar et al., 2019).

### 3.9 PTMs and epigenetic modifications

Epigenetic modifications and PTMs regulate gene expression at the transcriptional and post-transcriptional levels, respectively; however, their molecular mechanisms are highly interconnected, and act synergistically to maintain normal cellular homeostasis. PTMs can influence epigenetic states by regulating the activity and abundance of DNA methyltransferases and enzymes involved in histone modifications. Under normal conditions, UHRF1 functions as an E3 ubiquitin ligase involved in histone modification; however, upon DNA damage, phosphorylation of its Ser108 promotes its ubiquitination and mediates protein degradation, ultimately disrupting normal gene expression (Mancini et al., 2021; Chen H. et al., 2013). In addition, epigenetic modifications can also reciprocally reshape PTM networks by altering the gene expression of relevant enzymes. UBE3A, another E3 ubiquitin ligase, exhibits monoallelic expression under non-coding RNA-mediated genomic imprinting, with the paternal allele silenced, ultimately limiting its activity and regulatory capacity (LaSalle et al., 2015). Together, these mechanisms participate in maintaining intracellular homeostasis and regulating protein function and gene expression, forming a dynamic regulatory network.

## 4 Epigenetic modifications and HUA, gout

### 4.1 Methylation and HUA, gout

Methylation, as an important epigenetic modification, contributes to the progression from HUA to gout and drives inflammatory progression. Yang et al. (2024) found that *GLUT9* mRNA degradation increased when the expression of METTL14 increased in mouse renal epithelial cells. Subsequently, they used MeRIP to detect the level of m6A modification in the 3′UTR of this mRNA across cells with varying METTL14 expression, finding that m6A levels increased with METTL14 expression. These findings indicate that METTL14-mediated methylation of *GLUT9* mRNA inhibits GLUT9 expression and reduces UA reabsorption. In addition, Tin et al. (2021) in a multi-ancestry population study found that 99 CpG methylation sites were associated with SUA levels. Among them, hypermethylation of the *GLUT9* promoter downregulated its expression, reducing UA excretion. Besides the transport process, UA production is also regulated by methylation. Kimball et al. (2019) found that in bone marrow-derived macrophages (BMDMs) from mice with a defect in the methyltransferase setdb2, the repressive H3 lysine 9 trimethylation modification (H3K9me3) at the *XDH* promoter was reduced, thereby relieving repression of XDH and upregulating its synthesis and activity, ultimately promoting UA synthesis.

Furthermore, DNA methylation can also modulate the inflammatory response in gout, thereby accelerating its progression. Tseng et al. (2020) found that seven differentially methylated CpG sites associated with the IL-1β signaling pathway were identified in gout patients using genome-wide and methylation-wide analyses, and that these alterations were associated with gout inflammation. Further studies showed that abnormal methylation in the promoters of seven genes, including *INSIG1*, *MAPK8*, and *RPTOR*, can enhance IL-β signaling and accelerate the progression from HUA to gout. Wang et al. (2020) utilized BeadChip arrays to analyze the methylation of monocytes in the peripheral blood of gout patients. The results showed that 5,438 differentially methylated sites were present in gout patients and were significantly enriched in genes such as *GLUT9*, *PRKAG2* and *MEF2C*. These sites were associated with key pathways such as Th17 differentiation, B/T cell receptor signaling, and AMPK-related pathways. This suggests that abnormal DNA methylation may indirectly promote the activation of the NLRP3 inflammasome and IL-1β through remodeling immune networks, thereby contributing to gout pathogenesis.

In addition to mediating the progression from HUA to gout, methylation at specific sites is associated with individual susceptibility. Using quantitative methylation-specific polymerase chain reaction (qMSP) to determine DNA methylation levels in the peripheral blood of 57 male gout patients compared to 103 healthy men, Ying et al. (2019) found that *COMT* methylation was significantly lower in gout patients than in healthy men, which suggests that low *COMT* methylation may be associated with increased susceptibility to gout in males.

### 4.2 Kla and HUA, gout

Studies on the regulation of UA transporters and macrophage inflammation by Kla suggest that it is closely associated with the progression from HUA to gout.

Lv et al. (2026) investigated the renal protective effects of sea cucumber peptides by establishing mouse models of HUA nephropathy and UA-induced HK-2 cell models. Immunofluorescence, Western blotting (WB), and qRT-PCR analyses revealed that under HUA conditions, lactate accumulation in renal tissue was significantly increased, and H3Kla levels were elevated at multiple sites, including H3K14 and H3K19. Concurrently, URAT1 and GLUT9 expression increased, while ABCG2 expression decreased. These findings suggest that H3Kla may reduce UA excretion by regulating the expression of UA transporters, thereby elevating SUA.

Furthermore, while there is currently no direct evidence indicating that Kla promotes UA deposition, existing studies suggest that it may be involved in the inflammatory response following MSU crystal deposition. Zhang D. et al. (2019) systematically analyzed the expression of Kla and its transcriptional regulation in LPS and interferon-γ (IFN-γ) induced mouse macrophages using RNA-seq, ChIP-seq, and *in vitro* transcription assays. They found that during the late stages of macrophage activation, the levels of H3Kla were significantly elevated, and the expression of genes associated with tissue repair, such as *Arg1*, increased. Further experiments involving Ldha deficiency, glycolysis inhibitors, and exogenous lactate intervention demonstrated that Kla drives the transition of macrophages from an early pro-inflammatory state to a late-stage homeostatic repair state, suggesting that dysregulated Kla may be involved in the regulation of gout inflammation. Therefore, Kla may serve as a critical epigenetic link connecting UA metabolism and inflammatory imbalance, providing a potential target for mechanistic studies and therapeutic intervention.

### 4.3 Kcr and HUA, gout

Existing research on the role of Kcr in the progression from HUA to gout have primarily focused on how it modulates inflammation via regulation of the NLRP3 inflammasome and IL-1β.

Zheng et al. (2024) investigated the mechanisms underlying fluoride-induced renal immune-inflammatory damage using qPCR, WB, and immunoinflammatory phenotyping. They found that fluoride treatment significantly activated the NLRP3 inflammasome and was accompanied by elevated levels of inflammatory cytokines such as IL-1β. Concurrently, the levels of H2BK12cr and H4K8cr decreased, while decrotonylases such as SIRT1 and HDAC3 increased, suggesting that dysregulated Kcr may contribute to the development of gouty inflammation by regulating the activity of the NLRP3 inflammasome. Zou et al. (2022) modulated Kcr levels in models of neuropathic pain and crotonyl-CoA intervention models to investigate its role in neuropathic pain. They found that upregulation of Kcr promoted macrophage activation and increased the expression of inflammatory cytokines such as IL-1β. In contrast, inhibition of crotonyltransferases significantly attenuated these effects. Furthermore, Li L. et al. (2024) found that acetyl-CoA synthetase (ACSS2) increases H3K9cr and enhances its enrichment in the *Il1b* and *Il1r1* promoter regions, thereby promoting IL-1β expression. It can therefore be inferred that Kcr promotes the activation of inflammatory cells and the expression of inflammatory cytokines, thereby exacerbating gout-related inflammation.

## 5 PTMs and HUA, gout

### 5.1 Phosphorylation and HUA, gout

Protein phosphorylation plays a crucial role in the progression from HUA to gout by regulating multiple processes, including the specific transport and metabolism of UA, as well as inflammatory responses.

Under normal conditions, YAP1 acts as a transcription factor that promotes the expression of ABCG2 and OAT3 and maintains UA homeostasis. Han et al. (2024) investigated the role of WWC1 using gene silencing, overexpression, and WB in UOX knockout rats, HUA mice, and cell line models. The results showed that WWC1 upregulation in the renal tubular epithelium can promote YAP1 phosphorylation and subsequent degradation via the Hippo pathway, thereby decreasing the expression of ABCG2 and OAT3, and ultimately inhibiting UA excretion. (Li L. et al., 2024) performed WB on renal tissues from mice with HUA nephropathy that underwent sleeve gastrectomy (SG) or sham surgery. SG significantly increased the levels of nuclear factor erythrocyte-related factor 2 (Nrf2), adenosine monophosphate-activated protein kinase (AMPK), and ABCG2 in the kidneys of these mice. Further *in vitro* studies confirmed that AMPK activation increased ABCG2 expression, whereas Nrf2 knockdown significantly downregulated ABCG2 expression, indicating that AMPK activation and Nrf2 phosphorylation promote UA excretion by upregulating ABCG2.

In addition to UA metabolism, protein phosphorylation also regulates immune and inflammatory responses. Badii et al. (2025)examined the phosphorylation level of signal transducer and activator of transcription 1 (STAT1) in human peripheral blood monocytes by flow cytometry. They found that pretreatment with MSU followed by stimulation with LPS + MSU significantly reduced STAT1 phosphorylation in classical monocytes and HLA-DR + monocytes. Further analysis indicated that high concentrations of UA may suppress IFN-I-related signaling pathways by downregulating STAT1 phosphorylation in monocytes, thereby disrupting inflammatory homeostasis and ultimately amplifying gout inflammation. Previous studies have shown that the onset of gout is associated with the release of inflammatory mediators from neutrophils and ROS from macrophages (Ak and F, 2017). Furthermore, Futosi et al. (2023) used a cytokine array assay to detect the levels of neutrophil activation and inflammatory mediator release induced by MSU crystals in wild-type mice and some Src family tyrosine kinase-deficient (Hck^−/−^Fgr^−/−^Lyn^−/−^) mice. The results revealed that in deficient mice, the neutrophil response was significantly attenuated or even absent, and ROS production by macrophages was reduced. These results indicate that the amplification of MSU crystal-induced inflammation is closely linked to tyrosine phosphorylation mediated by the Src kinase family.

### 5.2 Glycosylation and HUA, gout

Protein glycosylation drives the progression from HUA to gout by regulating the intracellular localization of UA transporters and inflammatory pathways.

Regarding UA metabolism, Hou et al. (2019) conducted a comprehensive analysis of IgGn-glycans, ROC curves, and correlations in a cohort of 635 participants (208 males and 427 females) recruited in Beijing, finding that glycan peaks (such as GP1, GP6, GP4, GP11) were positively correlated with SUA. A model combining GP9, GP10, BMI, and sex yielded an area under the ROC curve of 0.849 for distinguishing between healthy individuals and those with HUA. This suggests that abnormal protein glycosylation is closely associated with UA metabolic dysregulation, and IgG glycosylation may serve as a potential biomarker for HUA. Building on this, further studies at the molecular level have revealed the regulatory role of glycosylation in UA transporters, Mandal and Mount (2019) found that the molecular weights of GLUT9 and ITM2B decreased after digestion with N-glycosidases, indicating that both are N-glycosylated proteins. Subsequently, WB analysis confirmed that the N-glycosylation site of GLUT9a and GLUT9b is located at Asn-90 and Asn-61, respectively. Notably, when these sites were mutated, ITM2B significantly inhibited GLUT9-mediated UA transport.

Glycosylation can also influence the intracellular processing and localization of UA transporters. When analyzing the glycosylation kinetics of ABCG2 and its different variants (Q141K, M71V) after release from the endoplasmic reticulum in cells, Bartos and Homolya (2021) found that the two variants showed aberrant glycosylation. They then labeled ABCG2 and its variants and tracked their intracellular trafficking with ER-Tracker Red and anti-giantin immunostaining. The results showed that, compared with ABCG2, only a small portion of Q141K and M71V variants were successfully transported to the cell surface via the Golgi apparatus to perform their normal functions, thereby limiting UA excretion. Similarly, studies have shown that an abnormal glycosylation process of uromodulin (UMOD), which is a secreted protein, reduces its localization at the cell surface, thereby limiting UA excretion (Vylet'Al et al., 2006).

Regarding gout-related inflammation, Han et al. (2026) used an LPS/MSU-induced THP-1 model and validated the findings in clinical samples. They found that the glycosyltransferase B3GALT2 is downregulated in gout patients, but its upregulation significantly inhibits NLRP3 inflammasome activation and IL-1β release. Furthermore, Qadri et al. (2018) found that, following MSU crystal stimulation and *in vivo* intervention in human THP-1 macrophages, Prg4^+/+^ and Prg4^−/−^ mouse peritoneal macrophages, and a rat model of MSU arthritis, the glycoprotein PRG4 inhibits MSU phagocytosis and the subsequent activation of the NLRP3 inflammasome and IL-1β release. These findings suggest that protein glycosylation dysregulation may contribute to the amplification of gout inflammation.

### 5.3 Ubiquitination and HUA, gout

The effects of protein ubiquitination on the progression from HUA to gout are primarily attributed to its promotion of MSU crystal-mediated inflammatory cascades, while also regulating UA metabolism.

Woodward et al. (2013) found that a mutation at position 141 in ABCG2, where glutamine is replaced by lysine, enhances ubiquitination-dependent proteasomal degradation of ABCG2, leading to reduced membrane expression and, ultimately, decreased UA excretion. Singh et al. (2021) investigated the intracellular signaling mechanism activated by MSU crystals and found that MSU crystals induce inflammation via TAK1-dependent signaling. Further studies showed that MSU crystals can enhance TAK1 kinase activity by binding to its active site and activating K^63^-linked polyubiquitination of TAK1, thereby promoting the formation of the downstream IRAK1/TRAF6/TAK1 complex, and ultimately amplifying the IL-1β-related inflammatory cascade.

In addition, Kim et al. (2016) analyzed the expression and ubiquitination levels of P62 protein in RAW264.7 cells treated with MSU crystals using WB as well as immunoprecipitation analysis, finding that these levels increased in the early stage of treatment. Further analysis of the expression and ubiquitination levels of apoptotic molecules, such as caspase-9 and caspase-3, in macrophages with and without P62 siRNA transfection revealed that the expression and ubiquitination levels of these apoptotic factors increased in non-transfected cells but decreased in P62-silenced cells. These findings indicate that MSU crystals can induce the ubiquitination and activation of apoptotic factors such as caspases through the ubiquitination of P62, thereby promoting macrophage apoptosis and IL-1β production. Juliana et al. (2012) reported that the NLRP3 inflammasome, which was originally ubiquitinated and inactive, underwent deubiquitination and activation after stimulation with activators such as LPS and ATP. Treatment with deubiquitination inhibitors, however, reduced the activity of the NLRP3 inflammasome, suggesting that the activation of the NLRP3 inflammasome caused by MSU crystal deposition is dynamically regulated by ubiquitination and deubiquitination. A later study showed that tumor necrosis factor receptor-associated factors (TRAF) also negatively regulate the activation of the NLRP3 inflammasome by inhibiting linear ubiquitination of ASC, thereby limiting the onset of gout (Mirzaesmaeili et al., 2023).

It is worth noting that ubiquitination dysregulation may also contribute to the development of HUA-related complications. Zhao H. et al. (2022) found that the degradation of IRS2 may be involved in HUA-induced diabetes based on RT-PCR analysis of insulin signaling-related genes. Subsequently, GO and KEGG analyses identified specific processes involving proteins interacting with IRS2 under HUA conditions. The results showed that E3 ligases involved in the ubiquitination process, the degradation of cyclins, and the ubiquitin-mediated degradation of Cdc25A all interact with IRS2, suggesting that protein ubiquitination may accelerate the progression from HUA to diabetes by regulating IRS2.

### 5.4 Acetylation and HUA, gout

Similar to protein ubiquitination, the role of protein acetylation in the progression from HUA to gout has also been extensively studied at the inflammatory level. However, Wang et al. (2016) conducted WB and immunoprecipitation analyses on HUA mice and Caco-2 cells induced by potassium oxonate combined with yeast polysaccharides and found that SIRT1 can deacetylate PGC-1α and activate the PGC-1α/PPARγ-ABCG2 pathway, thereby upregulating gastrointestinal ABCG2 expression to promote UA excretion. Misawa et al. (2015) explored the effect of resveratrol on NLRP3 inflammasome activation, and finding that resveratrol can alleviate the MSU crystal-induced NLRP3 activation and associated inflammatory damage by inhibiting the accumulation of acetylated tubulin, thereby blocking ASC-NLRP3 interaction and complex formation.

Lv et al. (2023) measured the expression level of Sirt3 (a deacetylase) in PBMCs of gout patients, and found that compared with healthy controls and patients with intermittent gout, patients with gouty attacks had the lowest expression level of Sirt3 while the highest level of acetylation of mitochondrial proteins. Further analysis of the gene expression profiles in Sirt3 wild-type or Sirt3-deficient BMDMs treated with palmitic acid or MSU crystals revealed that Sirt3 deficiency significantly upregulated the expression of inflammation-related genes such as *Acod1*, *Nlrc3*, *Itgb7*, and *Ccl2*, particularly *Acod1*. Sirt3 deficiency also enhanced ROS accumulation in mitochondria and NF-κB activation, and NF-κB inhibitors attenuated the upregulation of Acod1 expression. These findings suggest that Sirt3-mediated deacetylation negatively regulates the NLRP3/IL-1β inflammatory pathway via the mtROS/NF-κB axis.

### 5.5 Succinylation and HUA, gout

Emerging evidence suggests that succinylation contributes to the initiation and progression of gout by regulating inflammatory pathways.

Huang et al. (2026) investigated the role of the succinyltransferase KAT2A in the development of eclampsia using immunoprecipitation, WB, and CHX-chase assays, and found that KAT2A enhances the protein stability of NLRP3 by promoting the succinylation of its K21 site, thereby upregulating the release of inflammatory cytokines such as IL-1β and IL-18. Consequently, it can be inferred that NLRP3 succinylation may contribute to the development of gout by stabilizing NLRP3 and promoting inflammasome assembly. The binding of NIMA-related kinase 7 (NEK7) to NLRP3 is critical for inflammasome activation, whereas Wu and Ye (2024) examined the levels of NEK7 succinylation and its interaction with NLRP3 in the podocytes of mice induced by high-glucose conditions and found that SIRT5-mediated desuccinylation of NEK7 inhibits the binding of NEK7 to NLRP3, thereby suppressing the activity of the NLRP3 inflammasome. Thus, NEK7 succinylation indirectly promotes the development of gouty inflammation by enhancing the function of the NLRP3 inflammasome.

Tannahill et al. (2013) established LPS-stimulated BMDMs and a mouse inflammatory model, and analyzed succinate accumulation and protein succinylation in the cells using metabolomics and LC-MS, while simultaneously assessing the expression of HIF-1α and IL-1β via WB and qPCR. The results showed that LPS treatment significantly increased intracellular succinylation levels and protein succinylation, while upregulating IL-1β expression; conversely, inhibiting succinate accumulation led to a decrease in IL-1β expression, suggesting that elevated succinate levels and the accompanying succinylation may accelerate the progression of gout by promoting IL-1β-mediated inflammatory responses.

## 6 Conclusion and prospect

**FIGURE 2:**
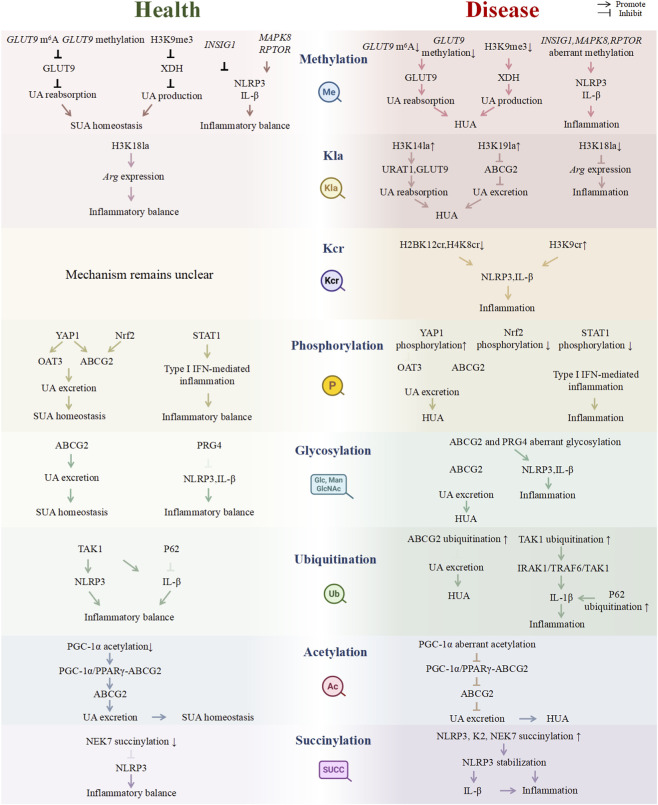
The effects of modifications on HUA, gout.

Current therapies for HUA and gout primarily focus on restoring UA homeostasis, regulating lipid metabolism, and modulating the gut microbiota; however, knowledge of the intracellular molecular events driving disease progression remains limited (Cheng et al., 2025; Ji et al., 2025; Li et al., 2025). This article briefly describes the pathogenesis of HUA and gout (Figure 1), as well as several common PTMs and epigenetic modifications, and, as summarized in Figure 2 and Table 1, reviews their complex relationships, revealing that these modifications participate in the mutual influence between HUA and gout at multiple levels by regulating gene expression, the conformation and function of UA transporters, and the NLRP3/IL-1β inflammatory axis. The pathways and molecules associated with PTMs and epigenetic modifications may serve as novel intervention targets, enabling targeted small-molecule interventions for HUA and gout. This opens new avenues for traditional therapeutic strategies and advances personalized medicine.

**TABLE 1:** The effects of modifications on HUA, gout.

Modification class	Modification type	Modification target	Direction of regulation	Mechanism	Biological effect	Phase	Ref.
Epigenetic modifications	Methylation	*GLUT9*	↑	↓GLUT9	UA excretion↓	HUA	Yang et al. (2024)
		*XDH*	↓	↑XDH	UA production↑		Kimball et al. (2019)
		*INSIG1, MAPK8, RPTOR*	--	↑IL-1β	Inflammation↑	Gout	Tseng et al. (2020)
		*GLUT9, PRKAG2, MEF2C*	--	↑NLRP3, IL-1β			Wang et al. (2020)
		*COMT*	↓	--			Ying et al. (2019)
	Kla	*H3*	*↑*	*↑*URAT1, GLUT9 ↓ABCG2	UA excretion↓	HUA	Lv et al. (2026)
			*↑*	*↑Arg1*	Inflammation↓	Gout	Zhang D. et al. (2019)
	Kcr	H2B, H4	↓	↑NLRP3, IL-β	Inflammation↑	Gout	Zheng et al. (2024)
		--	↑	↑IL-β, Macrophages			Zou et al. (2022)
		H3	↑	↑IL-β			Li L. et al. (2024)
PTMs	Phosphorylation	YAP1	↑	↓ABCG2, OAT3	UA excretion↓	HUA	Han et al. (2024)
		Nrf2	↑	↑ABCG2			Li Y. et al. (2024)
		STAT1	↓	↓IFN-I	Inflammation↑	Gout	Badii et al. (2025)
	Glycosylation	IgG	--	--	SUA↑	HUA	Hou et al. (2019)
		GLUT9	↑	↓GLUT9	UA excretion↓		Mandal and Mount (2019)
		ABCG2	--	↓ABCG2			Bartos and Homolya (2021)
		UMOD	--	↓UMOD			Vylet'Al et al. (2006)
		--	↑	↓NLRP3, IL-β	Inflammation↓	Gout	Han et al. (2026)
		PRG4	↑	↓MSU crystals, NLRP3, IL-β			Qadri et al. (2018)
	Ubiquitination	ABCG2	↑	↓ABCG2	UA excretion↓	HUA	Woodward et al. (2013)
		TAK1	↑	↑IRAK1/TRAF6/TAK1, IL-β	Inflammation↑	Gout	Singh et al. (2021)
		P62	↑	↑caspase, IL-β			Kim et al. (2016)
		NLRP3	↓	↑NLRP3			Juliana et al. (2012)
​	Acetylation	PGC-1α	↓	↑ABCG2	UA excretion↑	HUA	Wang et al. (2016)
		Tubulin	↓	↓NLRP3	Inflammation↓	Gout	Misawa et al. (2015)
		--	↑	↑NLRP3, IL-β	Inflammation↑		Lv et al. (2023)
	Succinylation	NLRP3	↑	↑NLRP3,IL-β,IL-18	Inflammation↑	Gout	Huang et al. (2026)
		NEK7	↑	↑NLRP3			Wu and Ye (2024)
		*--*	↑	↑IL-β			Tannahill et al. (2013)

Although some progress has been made in understanding the relationship between PTMs and epigenetic modifications in HUA and gout, limitations remain. On the one hand, most existing studies have focused on animal models and *in vitro* systems, with relatively insufficient clinical data; genetic polymorphisms across different species and the complex immune environment of the body limit clinical translation of these findings. On the other hand, the processes and cumulative effects of epigenetic modifications vary among individuals, and there is often cross-regulation between different modifications. Single-modality analyses are insufficient to elucidate the synergistic network underlying HUA-to-gout progression.

Therefore, future research should focus on the following areas. First, leveraging site-specific modifications and gene editing to further elucidate the roles of specific molecules in modification pathways in UA metabolism and inflammatory processes, with the aim of identifying interventions that are both targeted and safe. Second, integrating multi-omics strategies—including proteomics and metabolomics—to map the interactions between molecules involved in UA metabolism and inflammatory responses and various modifications. Third, transitioning from *in vitro* systems to *in vivo* models by developing models that better recapitulate disease progression and validating key targets using clinical evidence from patients, thereby improving clinical relevance.
